# Enhancement of Both Long-Term Depression Induction and Optokinetic Response Adaptation in Mice Lacking Delphilin

**DOI:** 10.1371/journal.pone.0002297

**Published:** 2008-05-28

**Authors:** Tomonori Takeuchi, Gen Ohtsuki, Takashi Yoshida, Masahiro Fukaya, Tasuku Wainai, Manami Yamashita, Yoshito Yamazaki, Hisashi Mori, Kenji Sakimura, Susumu Kawamoto, Masahiko Watanabe, Tomoo Hirano, Masayoshi Mishina

**Affiliations:** 1 Department of Molecular Neurobiology and Pharmacology, Graduate School of Medicine, University of Tokyo, Tokyo, Japan; 2 Department of Biophysics, Graduate School of Science, Kyoto University, and CREST, Japan Science and Technology Agency, Kyoto, Japan; 3 Department of Anatomy, Hokkaido University School of Medicine, Sapporo, Japan; 4 Department of Cellular Neurobiology, Brain Research Institute, Niigata University, Niigata, Japan; 5 Department of Molecular Function, Research Center for Pathogenic Fungi and Microbial Toxicoses, Chiba University, Chiba, Japan; Wellcome Trust Sanger Institute, United Kingdom

## Abstract

In the cerebellum, Delphilin is expressed selectively in Purkinje cells (PCs) and is localized exclusively at parallel fiber (PF) synapses, where it interacts with glutamate receptor (GluR) δ2 that is essential for long-term depression (LTD), motor learning and cerebellar wiring. Delphilin ablation exerted little effect on the synaptic localization of GluRδ2. There were no detectable abnormalities in cerebellar histology, PC cytology and PC synapse formation in contrast to GluRδ2 mutant mice. However, LTD induction was facilitated at PF-PC synapses in Delphilin mutant mice. Intracellular Ca^2+^ required for the induction of LTD appeared to be reduced in the mutant mice, while Ca^2+^ influx through voltage-gated Ca^2+^ channels and metabotropic GluR1-mediated slow synaptic response were similar between wild-type and mutant mice. We further showed that the gain-increase adaptation of the optokinetic response (OKR) was enhanced in the mutant mice. These findings are compatible with the idea that LTD induction at PF-PC synapses is a crucial rate-limiting step in OKR gain-increase adaptation, a simple form of motor learning. As exemplified in this study, enhancing synaptic plasticity at a specific synaptic site of a neural network is a useful approach to understanding the roles of multiple plasticity mechanisms at various cerebellar synapses in motor control and learning.

## Introduction

Various studies suggest the important roles of the cerebellum in the regulation of fine motor control and motor learning [1], [2]. The pattern of intrinsic neural connections in the cerebellum is known in considerable detail [3]. The wealth of knowledge of neural circuits in the cerebellum has led to the construction of models and theories of cerebellar functions [4]–[6]. These make the cerebellum an ideal system for studying the molecular and cellular mechanisms of brain function. The *N*-methyl-D-aspartate (NMDA) type of the glutamate receptor (GluR), a key molecule of synaptic plasticity and learning in the hippocampus and other forebrain regions, is absent in the cerebellar Purkinje cells (PCs). We found the δ subfamily of GluR by molecular cloning [7] and the second member of this subfamily, GluRδ2, is selectively expressed in cerebellar PCs [8], [9]. In PCs, GluRδ2 is exclusively localized at parallel fiber (PF)-PC synapses [10], [11]. Long-term depression (LTD) at PF-PC synapses, motor learning and motor coordination are impaired in GluRδ2 mutant mice [12]–[15]. In addition, a significant number of PC spines lack synaptic contacts with PF terminals and multiple climbing fiber (CF) innervation to PCs is sustained in GluRδ2 mutant mice [13], [16]–[18]. Furthermore, inducible ablation of GluRδ2 in the adult brain causes mismatching and disconnection of PF-PC synapses [19]. Thus, GluRδ2 plays a central role in the synaptic plasticity, motor learning and neural wiring of cerebellar PCs. There is no evidence for GluRδ2 channel activities, although lurcher mutation transformed GluRδ2 to constitutively active channels [20]. One possible signaling mechanism through GluRδ2 is by protein-protein interactions. Truncation of the carboxyl-terminal PSD-95/Discs large/zona occludens-1 (PDZ)-binding domain of GluRδ2 (T site) impairs LTD induction at PF-PC synapses and caused CF territory expansion, but had little effect on PF-PC synapse formation and elimination of surplus CFs at proximal dendrites of PCs [21]. Among PDZ proteins interacting with GluRδ2 at the T site, Delphilin appears to be interesting because of its selective expression in PCs except for a slight expression in the thalamus [22]. Within PCs, Delphilin is localized at PF synapses, but not at CF synapses. The characteristic expression pattern of Delphilin is reminiscent of GluRδ2. Here we report that Delphilin ablation results in the enhancement of both LTD induction at PF-PC synapses and optokinetic response (OKR) gain-increase adaptation, without affecting any detectable histological abnormalities. The phenotypes of Delphilin mutant mice are consistent with the idea that LTD induction at PF-PC synapses is a crucial rate-limiting step in OKR gain-increase adaptation, a simple form of motor learning.

## Methods

### Generation of Delphilin mutant mice

We isolated a mouse genomic clone carrying exon 2 and 3 of the *Delphilin* gene by screening a bacterial artificial chromosome library prepared from the C57BL/6 strain (Incyte Genomics, St. Louis, MO). The 34-bp *loxP* and 16-bp linker sequences were inserted into the *Avr*II site 93-bp upstream of exon 2, and the 1.9-kb DNA fragment carrying the 34-bp *loxP* sequence and *Pgk-1* promoter-driven *neo* gene flanked by two *frt* sites into the *Sph*I site 423-bp downstream of exon 3. Targeting vector pTVDEL1 contained exon 2 and 3 of the *Delphilin* gene flanked by *loxP* sequences, the 6.7-kb upstream and 2.3-kb downstream genomic sequences and 4.3-kb pMC1DTpA [23]. Homologous recombination in C57BL/6 embryonic stem cells and chimeric mouse production were carried out as described previously [19]. A chimeric mouse with the floxed *Delphilin* gene was mated to TLCN-Cre mice [24], [25], which were backcrossed 5 times to the C57BL/6 strain, to yield *Del^+/−^* mice. The *cre* gene was bred out and heterozygous Delphilin mutant mice were crossed with each other. Resulting homozygous mutant mice (*Del* ^−/−^) and wild-type littermates (*Del* ^+/+^) were used as mutant and control mice, respectively. The wild-type and mutant mice of 9 to 10 weeks old were used for subsequent analyses unless otherwise specified. The genotypes of mice were determined by polymerase chain reaction using primers 5′-GCTGGGAATGCAAGTCTGTT-3′ (DelP1), 5′-TGCGACACCACCTCGTCGAA-3′ (DelP2), and 5′-CTGACTAGGGGAGGAGTAGA-3′ (NeoR). Mice were fed *ad libitum* with standard laboratory chow and water in standard animal cages under a 12-h light: 12-h dark cycle. All animal procedures were approved by the Animal Care and the Use Committee of Graduate School of Medicine, the University of Tokyo (Approval # 1721T062), the Local Committee for Handling Experimental Animals in the Graduate School of Science, Kyoto University (Approval # H1804-12 and H1804-13), and the Animal Care and Use Committee of Hokkaido University (Approval # 06012).

### Western blot analysis

Whole homogenates were prepared from cerebella of mice at postnatal day 42 (P42) as described [26]. Western blot analysis was carried out as described [19]. Primary antibodies were guinea pig anti-Delphilin [22], rabbit anti-GluR2/3 (Upstate, Charlottesville, VA), rabbit anti-GluRδ2 [8], rabbit anti-postsynaptic density (PSD)-93 [27], rabbit anti-PTPMEG [28], rabbit anti-Synapsin I (Merck, Darmstadt, Germany) and rabbit anti-neuron specific enolase (NSE) [29]. Expression levels in the mutant mice were estimated as percentages of those in the wild-type mice using NSE as an internal standard.

### Histological analyses

Histological and electron microscopic analyses were carried out as described [19], [30]. Immunoperoxidase staining was carried out using guinea pig anti-Delphilin antibody. Double immunofluorescence was carried out using rabbit anti-calbindin [31], guinea pig anti-vesicular glutamate transporter 1 (VGluT1), guinea pig anti-vesicular glutamate transporter 2 (VGluT2), and rabbit anti-vesicular γ-amino butyric acid transporter (VGAT) [32] antibodies. To count PF-PC synapses on electron micrographs, 20 electron micrographs were taken randomly for each mouse from the molecular layer in the lobule IV/V at an original magnification of ×4,000 with an H-7100 electron microscope (Hitachi High-Technologies, Tokyo, Japan). Post-embedding immunogold analysis was carried out as described [22], [30] using rabbit anti-GluRδ2 antibody or the mixture of rabbit anti-GluR1, GluR2 and GluR3 antibodies [33].

### Electrophysiological analyses

Parasagittal cerebellar slices (250-µm thickness) were prepared from mice at P14-P18 unless otherwise stated. Whole-cell voltage-clamp recordings were performed on PCs in the II-VIII lobules of vermal region. A PC was whole-cell voltage-clamped with a patch pipette (2–3 MΩ) filled with the internal solution consisting of (in mM) 150 CsCl, 0.5 EGTA, 9 sucrose, 10 HEPES, 2 Mg-ATP (Sigma-Aldrich, St. Louis, MO) and 0.2 Na-GTP (Sigma-Aldrich), titrated to pH 7.3 with CsOH unless otherwise stated. The slices were continuously perfused with the oxygenated Krebs' solution containing (in mM) 124 NaCl, 1.8 KCl, 1.24 KH_2_PO_4_, 1.3 MgCl_2_, 2.5 CaCl_2_, 26 NaHCO_3_ and 10 glucose with 95% O_2_ and 5% CO_2_ at 22–24°C. Bicuculline (20 µM, Sigma-Aldrich) was added to suppress spontaneous inhibitory postsynaptic currents. Ionic currents were recorded with an EPC-9 or an EPC-10 amplifier (HEKA Elektronik, Lambrecht, Germany), and the signal was filtered at 1.5 or 2.9 kHz and digitized at 10 kHz. The membrane potential was held at −80 mV after compensation of the liquid junction potential unless otherwise stated.

To record miniature excitatory postsynaptic currents (mEPSCs), 1 µM tetrodotoxin (Wako Pure Chemical, Osaka, Japan) was applied to prevent action potential (AP) generation. The mean amplitude of mEPSC in a particular neuron was calculated from more than 300 mEPSCs, and the mean±SEM from 20 neurons are presented. The 10–90% rise time and the half-height width were measured in 10–11 mEPSCs in a PC and averaged, and the mean±SEM among 10 PCs was calculated. The metabotropic glutamate receptor type 1 (mGluR1)-mediated slow synaptic response was induced by repetitive stimulation of PFs (50 Hz, 1–20 times) in the molecular layer in the presence of 10 µM α-amino-3-hydroxy-5-methyl-4-isoxazolepropionic acid (AMPA) receptor blocker 2,3-dioxo-6-nitro-1,2,3,4-tetrahydrobenzo[f]quinoxaline-7-sulfonamide (NBQX) (Tocris Cookson, Bristol, UK) in addition to bicuculline. The CF response was induced by applying electrical stimulation (200 µs) to the granular layer near the soma of PC prepared from P22-P24 mice voltage clamped at −20 mV. In order to estimate the number of CF innervations, the intensity of stimulation was gradually increased from 0 V to 50 V by 3–5 V, and the number of amplitude steps in EPSCs was counted. It is known that most PCs are innervated by single CF at P22-P24. The Ca^2+^ current through voltage-gated Ca^2+^ channels was recorded by applying 20 ms depolarizing voltage pulses to a PC prepared from a P5 mouse in the presence of 10 mM tetraethylammonium chloride (Sigma-Aldrich), 1 mM 4-aminopyridine (Sigma-Aldrich) and 1 µM tetrodotoxin in addition to bicuculline and NBQX. Immature PCs were used in the Ca^2+^ current measurement to obtain a better voltage- and space-clamp condition. The series resistance compensation was optimized for Ca^2+^ current recording. The resting potential and AP were also recorded under the current-clamp condition with the K-gluconate internal solution in which CsCl and CsOH were replaced with K-gluconate and KOH, respectively. The series resistance compensation was optimized. PC firing frequency was measured under the cell-attached or current-clamp condition.

To monitor LTD, test pulses (1–10 V, 200 µs) were applied to PFs in the molecular layer at 0.05 Hz, except for the period of conjunctive stimulation. The intensity of stimulus was adjusted to evoke PF-EPSC whose initial amplitude was 100–200 pA. After stable recording for at least 7.5 min, the conditioning stimulation was applied to induce LTD. The conditioning stimulation was 200 ms depolarization of a PC to −20 mV coupled with the paired PF stimuli applied at 15 and 65 ms after the onset of depolarization. This conjunctive stimulation was repeated once, twice, 5 times, 10 times or 20 times at 1 Hz. In some experiments, 10 mM EGTA was added to the internal solution. Series resistance (10–30 MΩ) and input resistance were monitored every 2.5 min by applying a +10 mV, 80 ms voltage pulse to −70 mV. The data were discarded if the series resistance changed by more than 20% or the input resistance became <100 MΩ.

### OKR recordings

Eye movement recordings were performed by the video method as described [34], [35]. The sampling frequency of the image was 30 Hz. To induce OKR the screen with vertical black and white stripes (14°) that surrounds a mouse was rotated sinusoidally in light. The traces of eye velocity calculated from eye positions, and the stimulus (screen or turntable rotation) velocity were fitted with the respective sine curves by a least square method for at least successive 10 cycles except for the recording at 0.1 Hz (5 cycles or more). The gain of OKR was defined as the amplitude of fitted sine curve of eye velocity divided by that of stimulus. The negative value in phase indicates the lead of eye movement relative to the stimuli, and the positive value indicates the lag. Dynamic properties of OKR were measured twice and averaged values were used for the data analysis. To induce the adaptive change in OKR, the surrounding screen was rotated sinusoidally at 0.2 Hz, ±7.2° for 60 min each day. To prevent extinction of the learned response, the animals were kept in the dark between sessions. During the training paradigms, we made noises by clapping hands every 5 min in order to keep a mouse in an aroused state.

### Motor coordination test

Naive male mice were housed individually and were handled for ∼1 min a day for 7–10 days before behavioral tests. An animal was placed in the midpoint of a thin rod (TR-3002; O'Hara, Tokyo, Japan), and given six trials with 30-min inter-trial intervals. For a rotarod test, mice were habituated to an apparatus (RRSW-3002, O'Hara) by placing them on the rod rotating at 2.5 rpm (3×2 min sessions). An animal was placed on the rod rotating at 25 rpm, and given three trials with 45- to 60-min inter-trial intervals for 4 consecutive days.

### Statistical analyses

All behavioral experiments were performed in a blind fashion. Data were expressed as mean±SEM. Statistical analysis was performed using Student's *t* test, Mann–Whitney *U* test, Fisher's exact probability test or ANOVA with repeated measures as appropriate. Correlation analysis was done using Pearson's coefficient of comparison. Statistical significance was set at p<0.05.

## Results

### Cerebellar structure of mutant mice lacking Delphilin

**Figure 1:**
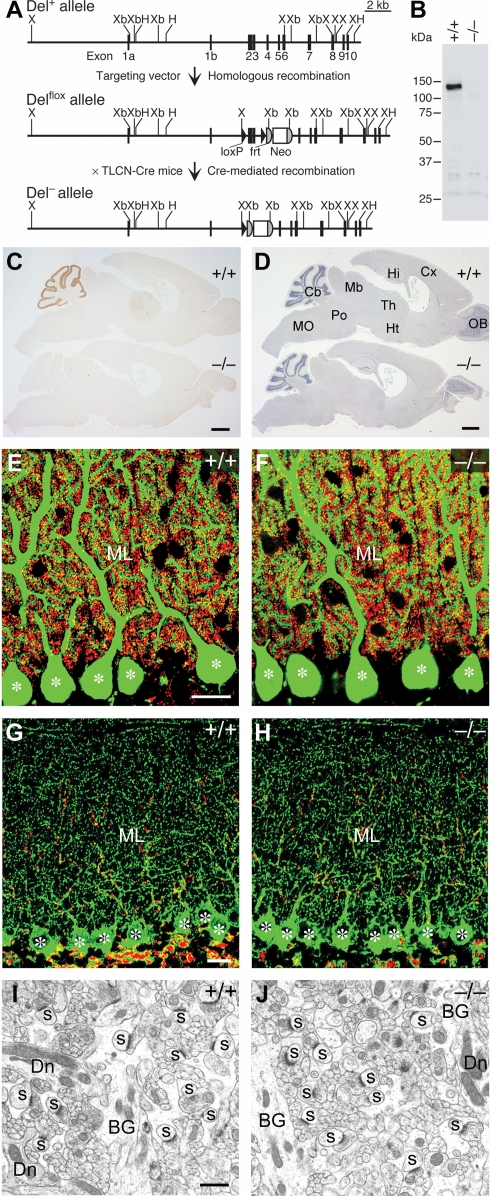
Generation and cerebellar structure of mutant mice lacking Delphilin. A, Schema of the *Delphilin* gene (*Del^+^*), floxed allele (*Del^flox^*), and null allele (*Del^−^*). Exon 3 encodes the PDZ domain of Delphilin. The *Del^flox^* allele contains two *loxP* sequences flanking exon 2 and 3 of the *Delphilin* gene and the *neo* gene flanked by two *frt* sequences. *Del^+/−^* mice were obtained by crossing *Del^+/flox^* mice with TLCN-Cre mice. Neo, neomycin phosphotransferase gene; H, *Hinc*II; X, *Xho*I, Xb, *Xba*I. B, Western blot analysis of Delphilin in cerebellar homogenates. C, Immunohistochemical analysis of Delphilin in the parasagittal brain sections. D, Hematoxylin staining of the parasagittal brain sections. Cb, cerebellum; Cx, Cerebral cortex; Hi, hippocampus; Ht, hypothalamus; Mb, midbrain; MO, medulla oblongata; OB, olfactory bulb; Po, pons; Th, thalamus. E,F, Double immunofluorescence for calbindin (green) and VGluT1 (red) in the cerebellar molecular layer of wild-type (E) and mutant (F) mice. Asterisks indicate the cell body of PCs. ML, molecular layer. G,H, Double immunofluorescence for VGAT (green) and VGluT2 (red) in the cerebellar molecular layer of wild-type (G) and mutant (H) mice. Asterisks indicate the cell body of PCs. ML, molecular layer. I,J, Electron micrographs of the cerebellar molecular layer of wild-type (I) and mutant (J) mice. BG, Bergmann glia; Dn, PC dendrite; s, PC spine in contact with PF terminals. Scale bars: C,D, 1 mm; E,G, 20 µm; I, 1 µm.

To examine the functional role of Delphilin in the cerebellum, we generated mutant mice lacking Delphilin (Fig. 1A). The Delphilin mutant mice grew and mated normally. Western blot analysis confirmed the absence of Delphilin of 135 kDa in the mutant mice (Fig. 1B). Strong immunohistochemical signals of Delphilin in the cerebellar molecular layer as well as faint signals in the thalamus attenuated in the mutant mice (Fig. 1C).

The cerebellum of the mutant mice exhibited normal foliation and laminated cortical structures (Fig. 1D). Double immunostaining for calbindin and VGluT1 revealed that PCs extended well-arborized dendrites studded with numerous spines (Fig. 1E,F), which were tightly associated with PF terminals (Fig. 1I,J). Immunostaining for VGluT2 and VGAT showed that the innervation patterns of CF and inhibitory terminals in the cerebellar molecular layer were comparable between the wild-type and mutant mice (Fig. 1G,H). In both genotypes, PC spines forming asymmetrical synapses were distributed in large numbers in the neuropil of the molecular layer (Fig. 1I,J). The cytoarchitecture and synaptic differentiation in the flocculus and paraflocculus were also indistinguishable between the wild-type and mutant mice (Fig. S1). The numbers of PF-PC synapses per 100 µm^2^ of the neuropil area were comparable between the wild-type (20.9±1.0, mean±SEM, n = 4) and mutant mice (20.7±0.5, n = 6; Mann–Whitney *U* test, p>0.05). Thus, Delphilin ablation exerted little effect on cerebellar histology, PC cytology and PC synapse formation.

### Expression and localization of GluRδ2

**Figure 2:**
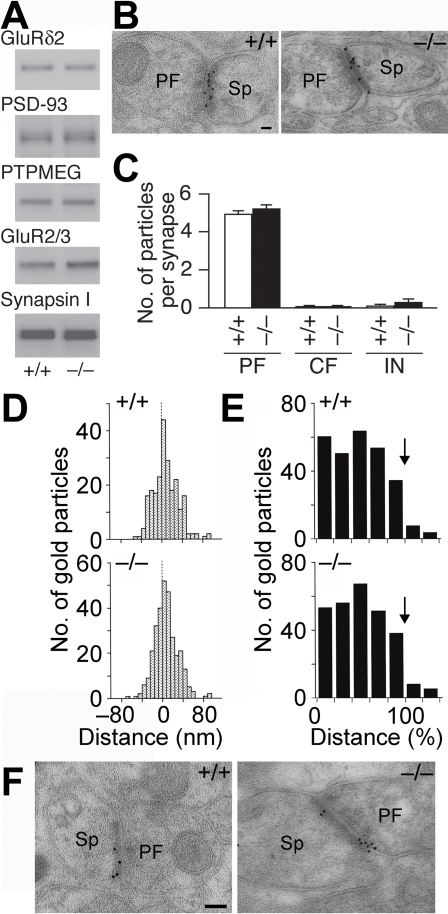
Expression and distribution of GluRδ2 at PF-PC synapses. A, Representative Western blots of GluRδ2, PSD-93, PTPMEG, GluR2/3 and Synapsin I in the cerebellum. B, Postembedding immunogold for GluRδ2 at PF-PC synapses. PF, parallel fiber; Sp, spine. C, The number of immunogold particles for GluRδ2 per profile of PF-PC synapses (+/+, n = 217; −/−, n = 179), CF-PC synapses (+/+, n = 12; −/−, n = 23) and IN-PC synapses (+/+, n = 9; −/−, n = 25). Data are expressed as mean±SEM. D, Perpendicular localization of GluRδ2 at PF-PC synapses. The distances from the midpoint of the postsynaptic membrane to the center of gold particles were grouped into 8-nm bins. E, Tangential localization of GluRδ2 at PF-PC synapses. The relative medio-lateral position of gold particles is indicated as the percentage of the distance from the center (0%) to the edge (100%) of the PSD. Arrows indicate the boundary of PSD. F, Postembedding immunogold for AMPA receptors at PF-PC synapses. PF, parallel fiber; Sp, spine. Scale bars: B,F, 100 nm.

Immunoblot analyses of the whole cerebellar homogenates showed that the amounts of GluRδ2 as well as PSD-93, PTPMEG and Synapsin I were comparable between the wild-type and mutant mice (n = 3 for each; Student's *t* test, p>0.2 in all cases), while those of anti-GluR2/3 antibody-immunoreactive AMPA receptor proteins were slightly increased in the mutant mice (p = 0.005, Fig. 2A). Both Delphilin and GluRδ2 are selectively localized at PF-PC synapses and interact with each other [13], [22]. We thus examined the effect of Delphilin ablation on the synaptic localization of GluRδ2 by the postembedding immunogold technique. In both genotypes, immunogold labeling of GluRδ2 was concentrated at PF-PC synapses (Fig. 2B). GluRδ2-particles were hardly found at CF-PC and interneuron (IN)-PC synapses (Fig. 2C). No significant differences were detected in labeling density between the wild-type and mutant mice in each type of PC synapses (Mann–Whitney *U* test, p>0.4 in all cases). In the perpendicular synaptic localization, gold particles for GluRδ2 peaked at 0–8 nm bin just postsynaptic from the midpoint of the postsynaptic membrane in both mice (Fig. 2D). In the tangential synaptic localization, gold particles were deposited uniformly along the postsynaptic membrane, except for the marginal 20% (80–100% bin) that showed a slight reduction (Fig. 2E). These results suggest that Delphilin ablation exerted little effect on the synaptic localization of GluRδ2. The synaptic distribution of AMPA receptors was also examined by the postembedding immunogold technique. Gold particles representing AMPA receptors were detected on the postsynaptic membrane of PF-PC synapses in both genotypes (Fig. 2F). When quantified, the number of gold particles of AMPA receptors per profile of PF-PC synapses in the mutant mice (6.4±0.3, n = 300 from 3 mice) was significantly larger than that in the wild-type mice (4.6±0.3, n = 300 from 3 mice; Mann–Whitney *U* test, p<0.001).

### Facilitation of LTD induction

There were no significant differences between the wild-type and mutant PCs in basal electrical properties including input resistance, resting potential, AP firing frequency, amplitude, threshold and half-height width (Table 1). The amplitude of mEPSCs was slightly larger in the mutant mice; however, the frequency and time course of mEPSCs were not significantly different between the genotypes (Table 1).

**Table 1:** Basal electrical properties of PCs in wild-type and mutant mice

Properties	Wild-type	Mutant
Resting potential, mV	−63±1 (10)	−61±2 (8)
Action potential		
Frequency, Hz	28±2 (31)	26±2 (32)
Amplitude, mV	62±2 (10)	60±2 (8)
Half-height width, ms	0.8±0.1 (10)	0.8±0.1 (8)
Threshold, mV	−41±0.6 (10)	−41±1.3 (8)
Input resistance, MΩ	294±29 (12)	313±45 (10)
mEPSC		
Amplitude, pA	11.3±0.4 (20)	12.8±0.5 (20)
Frequency, Hz	4.1±0.7 (20)	3.2±0.3 (20)
Half-height width, ms	12.9±0.8 (10)	12.8±0.7 (10)
10–90% rise time, ms	2.6±0.2 (10)	2.7±0.2 (10)
mGluR response		
Amplitude, pA	123±9 (6)	113±9 (6)
Half-height width, ms	737±121 (6)	989±206 (6)
10–90% rise time, ms	171±14 (6)	218±29 (6)
Ca^2+^ current at 0 mV, nA	2.0±0.3 (6)	1.8±0.2 (6)

Data are expressed as mean±SEM. Numbers in parentheses indicate the number of neurons. mGluR1 mediated synaptic response was induced by 10 pulses. There were no significant differences between wild-type and mutant PCs in basal electrical properties (Student's *t* test, p>0.1 in all cases) except for the amplitude of mEPSC (p = 0.02).

**Figure 3:**
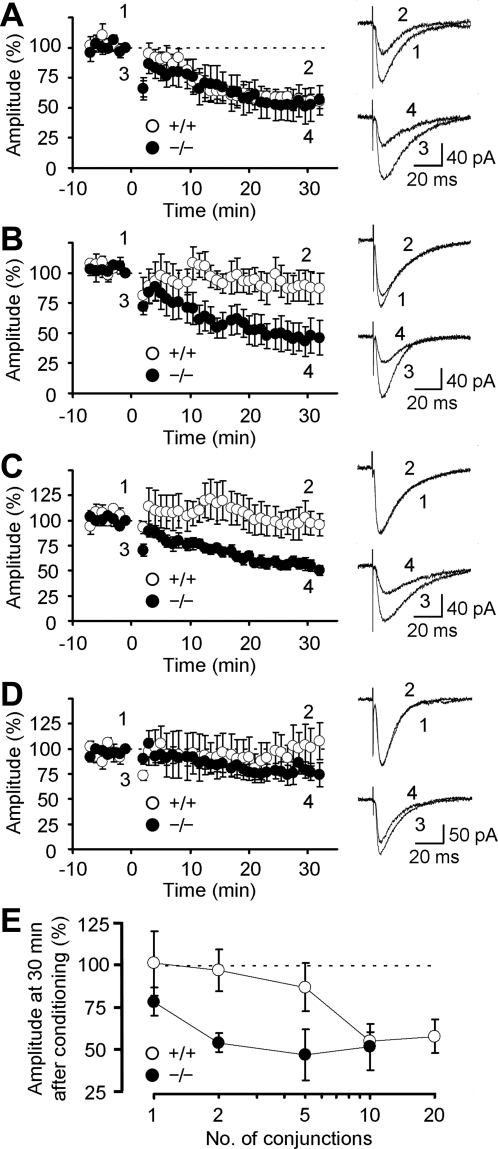
LTD at PF-PC synapses. A, Time courses of LTD at PF-PC synapses induced by 10-conjunction stimulation in wild-type and mutant mice. Representative PF-EPSCs recorded at the times indicated by numbers are shown on the right. The conditioning stimulation was applied at 0 min. The amplitude of PF-EPSC was normalized using the mean amplitude of EPSCs recorded for 1 min before the conditioning as the reference. B, Time courses of LTD induced by 5-conjunction stimulation. C, Time course of LTD induced by the 2 conjunctions. D, Time course of LTD induced by one conjunction. E, The percentile of depression 30 min after the conditionings was presented against the number of conjunctions. Data are expressed as mean±SEM.

In cerebellar slices of both genotypes, 10 paired-stimulations of PFs in conjunction with PC depolarization induced robust LTD (Fig. 3A). The amplitude of LTD measured 30 min after the induction in the mutant mice (52.5±13.7%, n = 7) was comparable to that in the wild-type mice (56.0±5.0%, n = 7; Student's *t* test, p = 0.82). The 5 conjunctions induced weak LTD in the wild-type mice (87.5±13.9%, n = 6). In contrast, robust LTD was induced in the mutant mice (47.8±14.9%, n = 6; p = 0.02; Fig. 3B). Further, 2 conjunctions failed to induce LTD in the wild-type mice (97.3±12.1%, n = 7). However, this weak conditioning successfully induced robust LTD in the mutant mice (55.0±5.4%, n = 10; p = 0.01; Fig. 3C). The amplitudes of LTD induced by the 2-conjunction stimulation in the mutant mice were comparable to those induced by the 10-conjunction stimulation in the wild-type or mutant mice (p = 0.90 and 0.87, respectively; Fig. 3E). Just one conjunction induced weak LTD in the mutant mice (78.9±8.3%, n = 6) but not in the wild-type mice (101.5±18.8%, n = 5; p = 0.28; Fig. 3D). These results do not necessarily mean that LTD can be induced by a single or a few conjunctions of CF and PF activities *in vivo*, because in the present experiments Cs^+^ was introduced into a PC that should have enhanced the Ca^2+^ influx. However, they suggest that LTD is induced relatively easily in the mutant mice *in vivo*. The time courses of LTD development (decrease in PF-EPSC amplitude) were similar irrespective of the number of conditioning conjunctions in both the wild-type and mutant mice. Together, these results suggest that Delphilin ablation facilitated LTD induction at PF-PC synapses with little effect on the saturation level of the amplitude.

**Figure 4:**
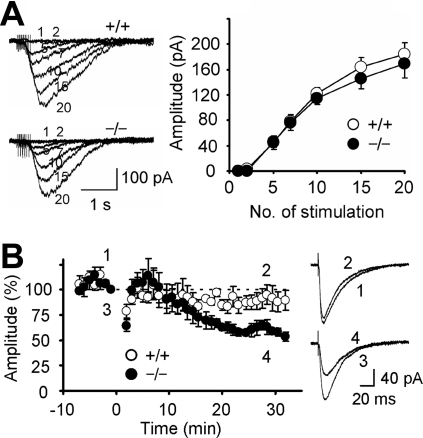
mGluR1-mediated synaptic response and effect of EGTA on LTD. A, mGluR1-mediated synaptic response induced in the presence of NBQX. PFs were repetitively (50 Hz, 1–20 pulses) stimulated. Representative traces and amplitudes of responses are presented. Each number beside traces represents the number of stimulation pulses. B, Time courses of LTD induced by 10-conjunction stimulation in PCs loaded with 10 mM EGTA. Representative PF-EPSCs recorded at the times indicated by numbers are shown on the right. The conditioning stimulation was applied at 0 min. Data are expressed as mean±SEM.

Ca^2+^ influx through voltage-gated Ca^2+^ channels, mGluR1 activation and AMPA receptor activation are required to induce LTD [36], [37]. The amplitudes of Ca^2+^ influx through voltage-gated Ca^2+^ channels for the two genotypes were similar (Table 1). A repetitive stimulation of PFs induces a slow inward current mediated by mGluR1 [38]. No significant difference was detected between the wild-type and mutant mice in terms of the amplitude and time course of the mGluR1-mediated slow synaptic response (Fig. 4A, Table 1). Cytosolic Ca^2+^ is necessary to induce LTD at PF-PC synapses [37]. When 10 mM EGTA was introduced into a PC, LTD was strongly suppressed in the wild-type mice (95.1±9.4%, n = 5) but only weakly in the mutant mice (63.1±7.2%, n = 5; Student's *t* test, p = 0.03; Fig. 4B). These results suggest that LTD was induced with less intracellular Ca^2+^ in the mutant mice.

**Figure 5:**
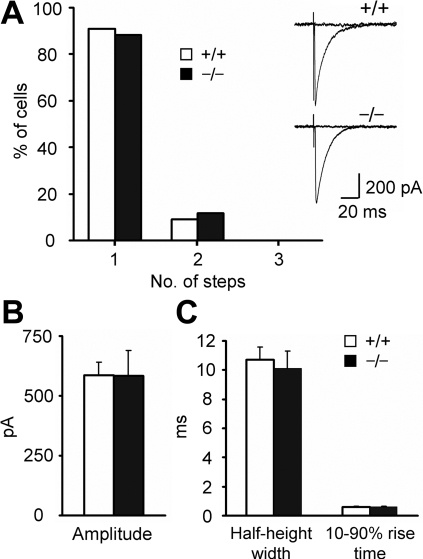
Synaptic responses at CF-PC synapses. A, The numbers of amplitude steps in CF-EPSCs (+/+, n = 22; −/−, n = 17). Representative CF-EPSCs are presented. B,C, The amplitude (B), half-height width and 10–90% rise time (C) of CF-EPSCs (+/+, n = 15; −/−, n = 10). Data are expressed as mean±SEM.

CF responses were recorded from P22-P24 mice, and the number of amplitude steps of EPSCs in response to the stimulation whose intensity was gradually increased was determined. CF responses with multiple amplitudes were limited in both genotypes (wild-type, 2 of 22; mutant, 2 of 17; Fig. 5A). This indicates that multiple innervations of CFs were rare in the mutant mice as in the wild-type mice (Fisher's exact probability test, p = 0.59). The amplitudes and time course of CF-EPSCs were also similar in the two genotypes (Student's *t* test, p>0.6 in all cases; Fig. 5B,C).

### Enhancement of OKR adaptation

**Figure 6:**
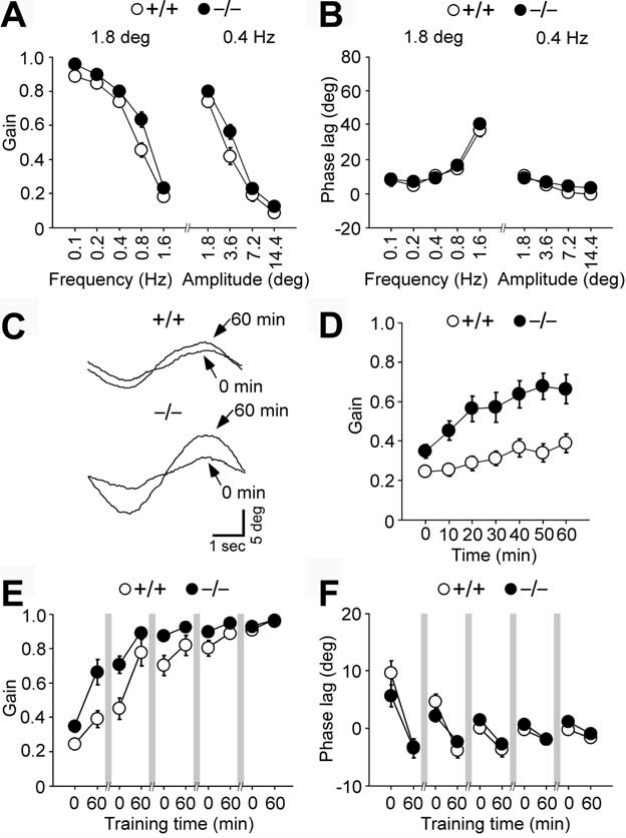
Dynamic properties and adaptive changes of OKR. A,B, Dynamic properties of OKR. The gain (A) and phase (B) values in wild-type (n = 14) and mutant (n = 13) male mice were measured during OKR. The peak amplitude of screen oscillation was fixed at 1.8°, or the frequency of screen oscillation was fixed at 0.4 Hz. C–F, Adaptive modification of OKR induced by a 60-min sustained sinusoidal screen oscillation at 0.2 Hz, ±7.2° in light over 5 days. Representative OKR traces before and after 60 min of sustained screen oscillation on day 1 (C). Changes in OKR gain during 60 min of sustained screen oscillation on day 1 in wild-type (n = 10) and mutant (n = 15) mice (D). Changes in OKR gain (E) and phase lag (F) over 5 days. There were rest periods for 23 h in the dark between training sessions as indicated by shaded bars. Data are expressed as mean±SEM.

To test the learning ability of Delphilin mutant mice, we examined OKR, the eye movement that follows the movements of large visual fields, and its adaptation. The cerebellar flocculus is involved in OKR adaptation, while vestibular nuclei play a central role in OKR [6]. OKR was elicited by sinusoidally oscillating a vertically striped screen surrounding an animal at 0.1–1.6 Hz, ±1.8–14.4° in light. The gain, which is the relative amplitude of eye movement against screen movement and the phase, which is the delay of eye movement from screen movement, were analyzed. The OKR gains of the wild-type and mutant mice decreased as the frequency of screen oscillation was increased at a fixed peak amplitude of 1.8° and as the angular amplitude of screen oscillation was increased at a fixed frequency of 0.4 Hz (Fig. 6A). Under these conditions, the mutant mice showed a slightly larger OKR gain than the wild-type mice (ANOVA with repeated measures, genotype effect, F_(1,25)_ = 6.0, p = 0.02). The OKR phase lags were less than 20° except at a frequency of 1.6 Hz (Fig. 6B). There were no significant differences in OKR phase lag between the two genotypes (genotype effect, F_(1,25)_ = 2.2, p = 0.15).

Continuous oscillation of the screen at 0.2 Hz, ±7.2° for 60 min induced an increase in OKR gains in both the wild-type and mutant mice (Fig. 6C,D). The adaptive increase in OKR gain in the mutant mice was significantly larger than that in the wild-type mice (genotype effect, F_(1,23)_ = 10.2, p = 0.004). The difference in basal OKR gain might have affected the learning process. However, there was no significant correlation between the basal OKR gain and the adaptive increase in OKR gain in each genotype (Pearson: wild-type, r = −0.30, p = 0.42; mutant, r = 0.46, p = 0.09). The adaptive decreases of OKR phase lag were similar for two genotypes (ANOVA with repeated measures, genotype effect, F_(1,23)_ = 0.35, p = 0.56). When the training was conducted for 5 consecutive days, the OKR gains of both the wild-type and mutant mice increased significantly with the number of training sessions (session effect, F_(9,207)_ = 82.1, p<0.001; Fig. 6E). On days 1 to 4, the mutant mice showed significantly larger gains than the wild-type mice (genotype effect, p = 0.008-0.04). However, the OKR gains of the wild-type mice became comparable to those of the mutant mice on day 5 (genotype effect, F_(1,23)_ = 0.28, p = 0.60). The OKR phase lag decreased with the number of training sessions in both genotypes (session effect, F_(9,207)_ = 25.8, p<0.001; Fig. 6F). There were no significant differences in phase lag between the two genotypes throughout the 5 training days (genotype effect, p = 0.08–0.64). Thus, Delphilin ablation augmented the adaptive increase in OKR gain but not the adaptive decrease in OKR phase lag.

**Figure 7:**
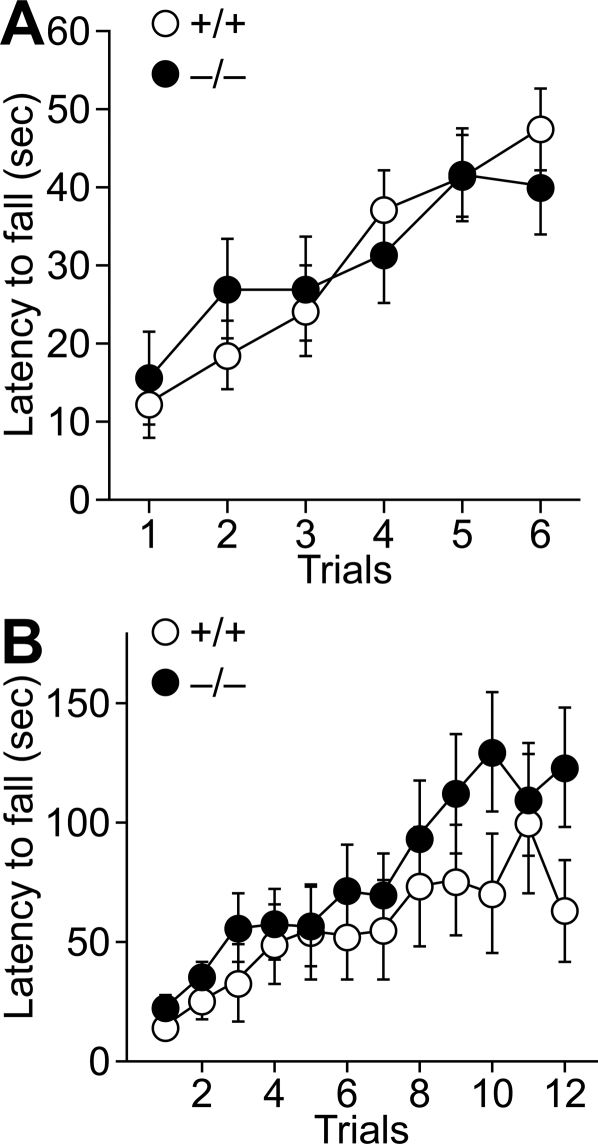
Motor coordination. A, The stationary horizontal thin rod test. Wild-type (n = 17) and mutant (n = 14) male mice were placed on the stationary horizontal thin rod and the time each mouse remained on the rod was measured. B, The rotating rod test. Rotarod performance of wild-type (n = 18) and mutant (n = 24) male mice. Retention time on the rotating rod at 25 rpm was measured. Data are expressed as mean±SEM.

Finally, the motor coordination of the mutant mice was examined. In the thin rod test, the retention times on a thin stationary plexiglass rod for the wild-type and mutant mice were indistinguishable (ANOVA with repeated measures, genotype effect, F_(1,29)_ = 0.002, p = 0.96; Fig. 7A). No significant differences in the retention time were observed on the rotating rod at 25 rpm between the two genotypes (genotype effect, F_(1,40)_ = 1.1, p = 0.29; Fig. 7B).

## Discussion

Here, we showed that Delphilin ablation at PF-PC synapses facilitates LTD induction at PF synapses and enhances OKR gain-increase adaptation without affecting any detectable histological abnormalities. This finding is compatible with the idea that LTD induction at PF-PC synapses is a crucial rate-limiting step in OKR gain-increase adaptation, a simple form of motor learning.

Examination of LTD under various stimulation conditions revealed that Delphilin ablation facilitated LTD induction at PF-PC synapses. On the other hand, the saturation levels of LTD amplitude for the wild-type and mutant mice were comparable. The time courses of LTD development after different numbers of conditioning conjunctions were also similar in both genotypes, implying that LTD expression itself proceeds normally in the mutant mice. Cumulative studies suggest that GluRδ2, mGluR1, AMPA receptors and Ca^2+^ are key mediators of LTD induction [36], [37]. However, Delphilin ablation appeared to exert little effect on the amount and localization of GluRδ2 and on the amplitude and kinetics of mGluR1-mediated slow synaptic responses. There were no significant differences between the wild-type and mutant PCs in basal electrical properties and the frequency and time course of mEPSCs although the amplitude of mEPSCs and the amount of AMPA receptors at PF-PC synapses were somewhat larger in the mutant mice. On the other hand, we observed that the Ca^2+^ requirement of LTD induction machinery was altered in the mutant mice, since 10 mM EGTA suppressed LTD strongly in the wild-type mice but only weakly in the mutant mice. The decreased dependence on the intracellular Ca^2+^ appears to be the main cause of the facilitation of LTD induction in the mutant mice.

Delphilin is distributed predominantly in cerebellar PCs and is localized selectively at PF synapses within PCs [22]. At PF-PC synapses, Delphilin binds to the carboxyl terminal of GluRδ2 that plays a central role in synaptic plasticity, motor learning and cerebellar wiring [13], [19], [39]–[41]. In contrast to the GluRδ2 mutant mice, the Delphilin mutant mice showed no detectable abnormalities in the cerebellar histology or morphology of PF synapses. In addition, Delphilin ablation exerted little effect on the expression and synaptic localization of GluRδ2. Consistently, the truncation of the PDZ-binding domain at the carboxyl terminal of GluRδ2 exerted little effect on the synaptic localization of receptor proteins, histological features and the fine structures of PF-PC synapses [21]. On the other hand, Delphilin ablation facilitated the induction of LTD, whereas LTD was impaired in the mutant mice carrying carboxyl-terminal truncated GluRδ2. It is likely that several domains for protein-protein interactions differentially mediate diverse GluRδ2 functions [19], [21], [42], [43] and multiple PDZ proteins interacting with the carboxyl terminal of GluRδ2, such as Delphilin, PSD-93, PTPMEG, nPIST and S-SCAM [22], [28], [44]–[46], may positively or negatively regulate LTD by mediating different downstream signaling. In fact, LTD was impaired in PTPMEG mutant mice [47]. The facilitation of LTD at PF-PC synapses in Delphilin mutant mice is reminiscent of the enhanced long-term potentiation (LTP) at hippocampal CA3-CA1 synapses in PSD-95 and synapse-associated protein 102 (SAP102) mutant mice [48], [49]. Delphilin, PSD-95 and SAP102 share similarities at the molecular levels–they are PSD proteins interacting with the carboxyl-terminal of glutamate receptors. PSD-95 ablation leads to the enhanced LTP under various stimuli whereas SAP102 mutant mice show the increase under more restricted conditions [48]–[50]. It is proposed that these synaptic membrane-associated guanylate kinase proteins couple the NMDA receptor to distinct signaling pathways [49], [51].

Functional impairment by manipulating molecules affecting plasticity or cellular signaling or both is one approach to clarifying their roles in motor control and learning [13], [52]–[54]. While the impairment at any site of the specific neural network may affect its function, the enhancement of plasticity at a specific site would affect the network function only if the site is a rate-limiting critical site. Thus, Delphilin mutant mice should be useful for this plasticity enhancement approach because Delphilin is selectively localized at PF-PC synapses and its ablation facilitates LTD induction with little effect on the maximal amplitude of LTD expression. OKR adaptation is accompanied by a change in Purkinje neuron activities in the cerebellar flocculus [55], and LTD has been implicated in the OKR adaptation [35], [56]. Since neurons are embedded in dynamic networks, some compensatory changes might occur in response to Delphilin ablation. Despite such possibility, the adaptive increase in OKR gain was significantly augmented in the Delphilin mutant mice. Thus, our results suggest the critical and rate-limiting role of LTD induction at PF-PC synapses in the neural network for OKR gain-increase adaptation, a simple form of motor learning. On the other hand, the adaptive decrease of OKR phase lag was unaltered by the mutation. In motor coordination tests, the performance of Delphilin mutant mice was also comparable to that of wild-type mice under the conditions used. Thus, the motor learning ability appears not to be generally facilitated in Delphilin mutant mice, although the possibility cannot be excluded that the conditions employed may be inadequate to detect the effect of the LTD modulation. It has been repeatedly reported that impairment of LTD is associated with motor learning deficits [13], [52]–[54]. However, there are controversial results showing that mice with diminished LTD have normal motor learning [57], [58]. Recent studies suggested that not gain-decrease vestibulo-ocular reflex (VOR) adaptation but gain-increase VOR adaptation depends on LTD and the dependence of VOR gain-increase adaptation on LTD differs depending on the frequency of training sinusoidal rotation [59], [60]. It was also reported that the ablation of fragile X mental retardation protein in PCs altered spine morphology and enhanced the maximum amplitude of LTD but attenuated eyeblink conditioning [61]. Thus, it appears that the contribution of LTD in diverse forms of motor control and learning is complicated. The critical synaptic sites in cerebellar neural networks may be variable depending on the types of diverse motor control and learning. In fact, various cerebellar synapses show adaptive plasticity [2], [36], [62]–[66]. Similar complications may underlie the fact that the overexpression of NMDA receptor 2B enhances hippocampal LTP and learning [67], whereas PSD-95 mutant mice show severe impairments in spatial learning and SAP102 mutant mice have mild impairments despite of enhanced hippocampal LTP [48], [49]. Further analyses will be required to clarify the issue.

The wealth of knowledge of the neural circuits makes the cerebellum an ideal system for studying the molecular and cellular mechanism of brain functions. Various cerebellar synapses show multiple forms of synaptic plasticity [2], [36], [62]–[66] and may play differential roles in diverse motor control and learning. As exemplified in this study, enhancing synaptic plasticity at a specific synaptic site of a neural network is a useful approach to understanding the roles of multiple plasticity mechanisms at various cerebellar synapses in motor control and learning.

## Supporting Information

Figure S1 Anatomical analysis in the flocculus and paraflocculus. A,B, Hematoxylin staining of coronal cerebellar sections from wild-type (A) and mutant (B) mice. Co, cochlear nucleus; Fl, flocculus; PFl, paraflocculus. C–F, Double immunofluorescence for calbindin (green) and VGluT2 (red) in the flocculus (C,D) and paraflocculus (E,F) of wild-type (C,E) and mutant (D,F) mice. Asterisks indicate the cell body of PCs. ML, molecular layer. G–J, Electron micrographs of the cerebellar molecular layer of the flocculus (G,H) and paraflocculus (I,J) of wild-type (G,I) and mutant (H,J) mice. s, PC spine in contact with PF terminals. Scale bars: A, 500 µm; C, 20 µm; G, 500 nm.
(9.87 MB TIF)
