# Supplementary figures and images for: Enhancement of Both Long-Term Depression Induction and Optokinetic Response Adaptation in Mice Lacking Delphilin
Source: PLoS One. 2008 May 28;3(5):e2297. doi: 10.1371/journal.pone.0002297 (PMC2386150; doi:10.1371/journal.pone.0002297)

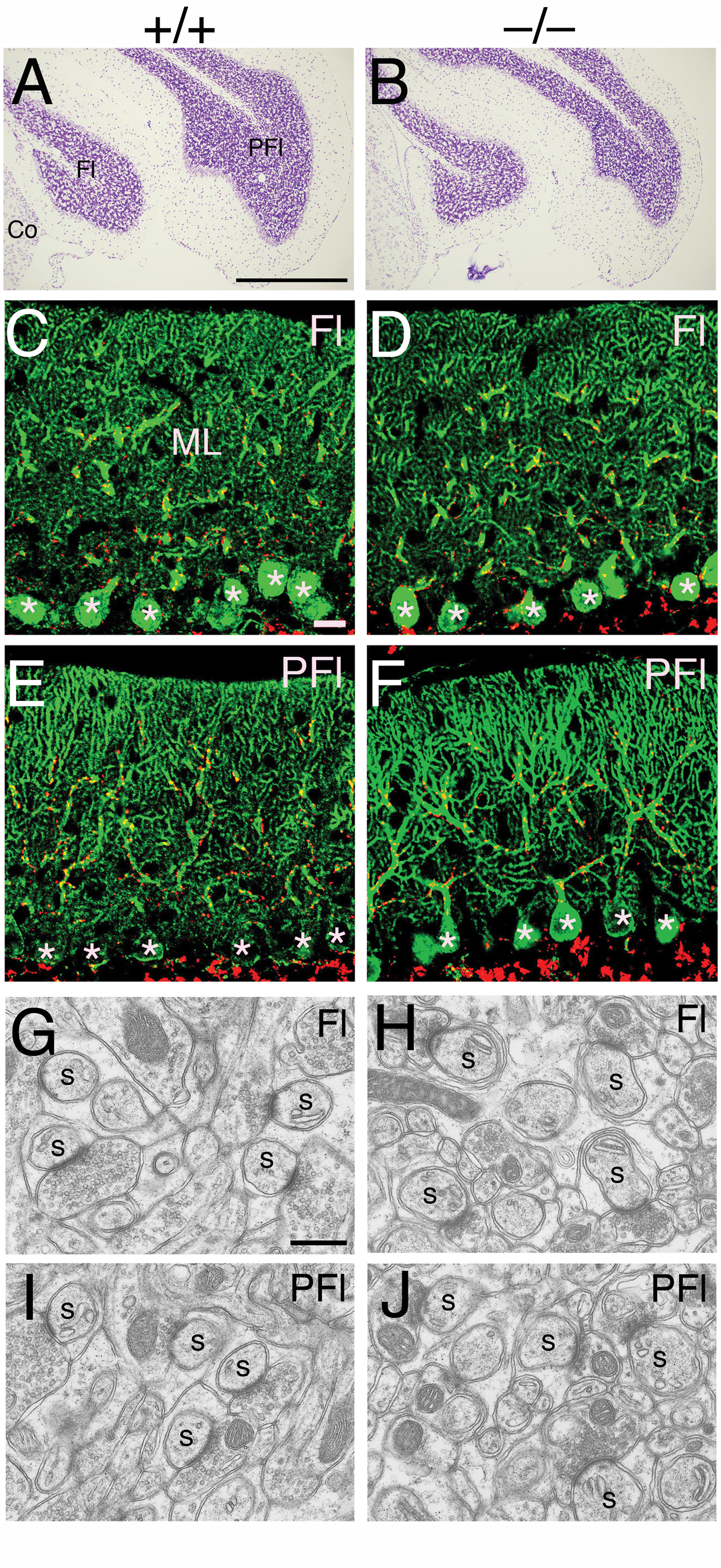

Supplement: Figure S1 — Anatomical analysis in the flocculus and paraflocculus. A,B, Hematoxylin staining of coronal cerebellar sections from wild-type (A) and mutant (B) mice. Co, cochlear nucleus; Fl, flocculus; PFl, paraflocculus. C–F, Double immunofluorescence for calbindin (green) and VGluT2 (red) in the flocculus (C,D) and paraflocculus (E,F) of wild-type (C,E) and mutant (D,F) mice. Asterisks indicate the cell body of PCs. ML, molecular layer. G–J, Electron micrographs of the cerebellar molecular layer of the flocculus (G,H) and paraflocculus (I,J) of wild-type (G,I) and mutant (H,J) mice. s, PC spine in contact with PF terminals. Scale bars: A, 500 µm; C, 20 µm; G, 500 nm. (9.87 MB TIF) [file pone.0002297.s001.tif]
